# Glycolytic reprogramming in cancer: immune crosstalk, nutrient competition, and supportive care perspectives

**DOI:** 10.3389/fimmu.2026.1862570

**Published:** 2026-06-08

**Authors:** Hui Wu, Yan Wang, Yu Tian, Shipin Feng, Xiongtao Yang, Xiaoli Yuan, Linlin Fan, Qiang Feng, Zhaoxia Liu, Qi Zhao

**Affiliations:** 1 Department of General Internal Medicine, Sichuan Clinical Research Center for Cancer, Sichuan Cancer Hospital and Institute, Sichuan Cancer Center, University of Electronic Science and Technology of China, Chengdu, China; 2 Department of Medical Oncology, Huizhou Central People’s Hospital, Huizhou, Guangdong, China; 3 Department of Pediatric Nephrology, Chengdu Women’s and Children’s Central Hospital, School of Medicine, University of Electronic Science and Technology of China, Chengdu, China; 4 Department of Surgical Ward 1 (Thoracic Surgery/Head and Neck Surgery), Sichuan Clinical Research Center for Cancer, Sichuan Cancer Hospital and Institute, Sichuan Cancer Center, University of Electronic Science and Technology of China, Chengdu, China

**Keywords:** cancer immunotherapy, glycolytic reprogramming, lactate, nutrient competition, tumor immune escape

## Abstract

Cancer-associated glycolytic reprogramming has long been recognized as a hallmark of malignant metabolism, yet its importance extends far beyond supporting tumor growth and biosynthesis. Increasing evidence indicates that enhanced glycolysis profoundly reshapes the tumor microenvironment by redistributing nutrients, increasing lactate accumulation, and creating acidic, immunosuppressive conditions. Through these changes, tumor glycolysis influences not only cancer cell survival but also the function of immune and stromal populations, thereby linking metabolic adaptation to immune escape and therapeutic resistance. In particular, excessive glucose consumption by tumor cells restricts nutrient availability for effector lymphocytes, whereas lactate and acidification impair T-cell fitness, promote suppressive myeloid phenotypes, and support fibroblast-mediated immunosuppressive niche formation. In parallel, glycolysis-associated signaling programs can directly regulate immune checkpoints such as PD-L1, further integrating tumor metabolism with immune evasion. These findings support a broader view of glycolysis as an organizer of tumor immune ecology rather than a purely tumor-intrinsic metabolic event. In this review, we discuss the molecular basis of glycolytic reprogramming, its role in immune crosstalk and nutrient competition, and its contribution to resistance against radiotherapy, chemotherapy, and immunotherapy. We further highlight emerging therapeutic opportunities, including direct glycolysis targeting, metabolism-directed nanomedicine, nutritional intervention, and metabolic engineering of immune cells. Together, these advances suggest that effective future strategies should combine tumor metabolic restriction with restoration of immune metabolic fitness.

## Highlights

- Glycolytic reprogramming actively organizes the tumor immune microenvironment rather than serving only as a metabolic hallmark of cancer cells.
- Glucose competition, lactate accumulation, and acidification jointly impair T-cell function while promoting myeloid and stromal immunosuppressive programs.
- Therapeutic strategies combining tumor glycolysis restriction with immune metabolic support may improve responses to cancer immunotherapy.

## 1 Introduction

Glycolytic reprogramming is widely recognized as one of the defining features of malignant transformation (1–3). However, its significance in cancer extends far beyond a simple increase in glucose consumption or lactate production. In tumor cells, enhanced glycolysis supports rapid proliferation by supplying not only ATP but also a broad range of biosynthetic intermediates required for nucleotide, amino acid, and lipid synthesis (4, 5). This metabolic configuration also facilitates redox balance and stress adaptation, thereby allowing cancer cells to survive under fluctuating oxygen and nutrient conditions. From this perspective, glycolytic reprogramming is not merely an energetic shortcut, but a flexible and actively regulated program that sustains tumor growth, plasticity, and survival. Importantly, the consequences of glycolytic reprogramming are not confined to tumor cells themselves. As cancer cells increase glucose uptake and channel metabolic flux toward lactate generation, they profoundly reshape the biochemical landscape of the tumor microenvironment (6, 7). Glucose availability becomes limited, lactate accumulates, extracellular pH declines, and the distribution of metabolic resources becomes increasingly uneven. These alterations create a competitive and often hostile environment for surrounding stromal and immune cells. Thus, tumor glycolysis should not be viewed as an isolated intracellular event, but rather as a microenvironment-shaping process with broad ecological consequences. This metabolic rewiring has particularly important implications for antitumor immunity. Immune cells, especially activated CD8+ T cells, also require substantial metabolic resources to sustain proliferation, effector cytokine production, cytotoxicity, and long-term function (8–10). When tumor cells establish a dominant glycolytic state, they do not simply strengthen their own fitness; they simultaneously impose metabolic stress on neighboring immune populations. In parallel, glycolysis-derived metabolites such as lactate act as signaling molecules that influence the differentiation, polarization, and suppressive activity of multiple immune and stromal cell types. In this way, glycolytic reprogramming shapes not only tumor cell behavior, but also immune cell fate and function (8). Accordingly, glycolysis should now be regarded as a central hub linking tumor metabolism, immunosuppressive microenvironment formation, and therapy resistance. Its role is no longer adequately captured by the classical view of the Warburg effect as a hallmark of cancer cell metabolism alone. Instead, glycolytic reprogramming must be understood as a systems-level process that integrates metabolic adaptation with immune regulation and therapeutic responsiveness. This broader perspective provides an essential conceptual foundation for understanding why targeting cancer metabolism may have profound immunological and translational implications.

The study of tumor immune escape has traditionally focused on immune checkpoints, immunosuppressive cytokines, defective antigen presentation, and the altered composition of immune infiltrates. These mechanisms remain fundamental to our understanding of how tumors evade immune surveillance. However, this framework is incomplete if tumor immunity is interpreted only through receptor–ligand interactions or immune cell abundance. Increasing evidence indicates that immune responses in tumors are also profoundly shaped by metabolic conditions, including nutrient availability, metabolite accumulation, and local bioenergetic stress (11–14). Within the tumor microenvironment, immune cells do not operate in metabolically neutral space. Instead, they must function in an environment where resources are continuously consumed, redistributed, and contested. Tumor cells with enhanced glycolytic activity rapidly deplete glucose, thereby restricting a key nutrient required for T-cell activation and effector differentiation (15). At the same time, excessive lactate production and acidification create conditions that impair immune cell proliferation, cytokine secretion, and cytotoxic activity. As a result, metabolic pressure becomes an underappreciated but decisive determinant of whether antitumor immunity is sustained or functionally exhausted. This metabolic reinterpretation is especially important because it helps explain why immune dysfunction in cancer often cannot be fully accounted for by checkpoint signaling alone. For example, even when immune cells are present within tumors, they may remain ineffective because the surrounding metabolic environment is incompatible with durable effector function. In other words, immune suppression is not only imposed through inhibitory receptors or suppressive cytokines, but also through deprivation, toxicity, and metabolic competition. Nutrient restriction and metabolite-mediated signaling therefore represent critical layers of immune regulation that intersect with, reinforce, and sometimes even precede canonical immune escape pathways (16). Among these metabolic factors, glucose deprivation, lactate accumulation, and extracellular acidification are particularly relevant in glycolysis-high tumors. Together, they translate a tumor-intrinsic metabolic phenotype into an immunosuppressive microenvironmental state. This conversion is conceptually important: it means that a shift in cancer cell metabolism can directly alter the functionality of T cells, myeloid cells, fibroblasts, and other components of the tumor ecosystem. Tumor immunity must therefore be reinterpreted through a metabolic lens, not as a replacement for immunological models, but as an essential extension of them. Such a perspective allows a more integrated understanding of how immune escape is established, maintained, and potentially reversed.

In this review, we focus on three closely connected dimensions of glycolytic reprogramming in cancer: immune crosstalk, nutrient competition, and therapeutic opportunities. Rather than treating glycolysis solely as a tumor-intrinsic metabolic hallmark, we examine it as a dynamic driver of communication between cancer cells and the surrounding microenvironment. This perspective is particularly important because the biological consequences of glycolytic reprogramming emerge not only from the metabolic state of tumor cells, but also from how that state reshapes the behavior of immune and stromal populations. First, we discuss immune crosstalk mediated by glycolytic activity and its metabolic byproducts. Special attention is given to how lactate, acidification, and glycolysis-associated signaling pathways influence myeloid cells, T cells, and stromal components such as cancer-associated fibroblasts(CAFs). Through these interactions, tumor glycolysis contributes to the establishment of an immunosuppressive niche that supports tumor progression and weakens antitumor immunity. We therefore consider glycolytic metabolism not only as a cellular program, but also as a mechanism of intercellular communication within the tumor ecosystem. Second, we examine nutrient competition as a central determinant of immune dysfunction in cancer. Tumor cells and immune cells share overlapping metabolic demands, particularly for glucose, yet they do not have equal access to metabolic resources within the tumor microenvironment (17). As tumors intensify glycolytic flux, they create conditions of nutrient scarcity and metabolic stress that compromise immune cell activation, persistence, and cytotoxic capacity. We highlight how this competition affects antitumor T-cell function and discuss emerging evidence that strengthening immune cell metabolic fitness may help overcome these limitations. Third, we review current and emerging therapeutic opportunities arising from this conceptual framework. These include direct targeting of tumor glycolysis, strategies to neutralize the immunosuppressive effects of lactate and acidosis, metabolic engineering of therapeutic immune cells, and rational combinations with immune checkpoint blockade or other anticancer therapies. By integrating mechanistic insights with translational advances, this review aims to show how understanding glycolytic reprogramming can provide new opportunities to improve cancer treatment. Overall, our goal is to present glycolytic reprogramming as a unifying framework that links tumor metabolism with immune suppression, microenvironmental competition, and therapeutic vulnerability. By focusing on the metabolic interactions between tumor cells, immune cells, and stromal cells, we seek to provide a more integrated view of how immune escape develops and how it may be more effectively targeted in the future.

## 2 Molecular basis of glycolytic reprogramming in cancer

### 2.1 Core metabolic features of the glycolytic phenotype

The glycolytic phenotype of cancer is characterized by coordinated metabolic adaptations that extend beyond a simple increase in glucose consumption. One of its most prominent features is enhanced glucose uptake, which is driven by increased expression and activity of glucose transporters and by the activation of intracellular pathways that favor continuous glucose influx (18). This augmented uptake provides tumor cells with a stable carbon source that supports both energy production and biosynthetic demand. In rapidly proliferating cancer cells, glucose is not merely oxidized for ATP generation but is preferentially directed into interconnected metabolic routes that sustain cellular growth, replication, and stress tolerance.

A second defining feature is the upregulation of key glycolytic enzymes and regulatory nodes, including hexokinase 2 (HK2), 6-phosphofructo-2-kinase/fructose-2,6-bisphosphatase 3 (PFKFB3), pyruvate kinase M2 (PKM2), lactate dehydrogenase A (LDHA), and pyruvate dehydrogenase kinase 1 (PDHK1) (19–23). Together, these molecules reinforce glycolytic flux at multiple levels. HK2 promotes the first committed step of glucose metabolism and is often linked to mitochondrial function and cell survival. PFKFB3 enhances the production of fructose-2,6-bisphosphate, thereby sustaining a high glycolytic rate. PKM2 supports metabolic flexibility by allowing glycolytic intermediates to accumulate upstream for anabolic use, whereas LDHA favors the conversion of pyruvate into lactate, enabling continued NAD+ regeneration under conditions of high glycolytic demand (24). PDHK1 further diverts pyruvate away from mitochondrial oxidation by inhibiting pyruvate dehydrogenase, thereby reinforcing glycolysis and limiting entry into the tricarboxylic acid cycle (25, 26).

As a consequence of this metabolic configuration, lactate production is markedly increased in many tumors. Lactate accumulation is not simply a passive byproduct of altered metabolism but a predictable outcome of sustained glycolytic flux combined with pyruvate diversion away from oxidative metabolism. The progressive export of lactate from tumor cells contributes to extracellular acidification, generating a locally acidic microenvironment. This acidification has broad biological effects, including the modulation of enzyme activity, extracellular matrix remodeling, invasion, angiogenesis, and intercellular communication. Thus, the glycolytic phenotype extends beyond intracellular metabolism and directly alters the biochemical properties of the tumor niche.

Another important feature of glycolytic reprogramming is the redistribution of glycolytic intermediates into biosynthetic and adaptive pathways. Intermediates derived from glycolysis can be shunted into the pentose phosphate pathway to support nucleotide synthesis and redox homeostasis, or into amino acid, lipid, and one-carbon metabolism to sustain biomass accumulation and cellular plasticity. This branching architecture allows glycolysis to function as a metabolic backbone for tumor expansion. At the same time, the glycolytic state provides adaptive advantages under hypoxic, inflammatory, and nutrient-variable conditions, enabling cancer cells to maintain viability and proliferative competence even when mitochondrial metabolism is constrained or fluctuating. Therefore, the glycolytic phenotype should be understood as an integrated metabolic program that simultaneously supports growth, survival, and environmental adaptation.

### 2.2 Oncogenic and regulatory networks sustaining glycolysis

The glycolytic phenotype in cancer is not a random consequence of rapid proliferation but the product of tightly coordinated oncogenic and regulatory networks. Among the most established drivers is hypoxia-inducible factor 1 alpha (HIF-1α), which plays a central role in adapting tumor metabolism to hypoxic and stress-associated conditions (27). HIF-1α transcriptionally activates multiple genes involved in glucose uptake, glycolytic flux, lactate production, and pyruvate diversion, thereby promoting a metabolic state that favors glycolysis even when oxygen is present (28–30). Through its induction of transporters and enzymes such as LDHA and PDHK1, HIF-1α helps tumor cells maintain ATP production, redox balance, and survival in hostile microenvironments. Importantly, HIF-1α-driven glycolysis is not only a survival mechanism but also a key contributor to microenvironmental acidification and immune suppression, linking hypoxic adaptation to broader tumor ecosystem remodeling.

Another major regulator is c-Myc, a pleiotropic oncogenic transcription factor that coordinates cell growth with metabolic demand. c-Myc enhances the expression of multiple genes involved in glucose uptake and glycolytic processing, while also supporting nucleotide, amino acid, and lipid biosynthesis (31, 32). In this way, c-Myc helps establish a metabolic architecture in which glycolysis is coupled to biomass production and proliferative expansion. Because c-Myc integrates metabolic reprogramming with cell cycle progression and stress adaptation, its activity places glycolysis at the center of oncogenic growth control. Emerging studies further indicate that c-Myc-dependent metabolic programs intersect with immune regulatory pathways, suggesting that glycolysis-promoting transcriptional networks may also influence immune checkpoint expression and therapeutic responsiveness (33–36).

In addition to classical transcriptional regulators, recent evidence has highlighted the importance of signaling and post-transcriptional mechanisms that sustain glycolysis. One representative example is the PRMT3/PDHK1 axis. By promoting PDHK1-dependent inhibition of mitochondrial pyruvate oxidation, this pathway reinforces glycolytic dependency and favors lactate-generating metabolism (37). Of particular relevance, such signaling circuits do not merely intensify tumor metabolism; they can also converge on immune escape mechanisms, including the upregulation of PD-L1. This observation is conceptually important because it illustrates that metabolic rewiring and immune regulation are not parallel phenomena, but increasingly appear to be mechanistically interconnected.

Non-coding RNA-mediated regulation represents another important layer of glycolytic control. The lncRNA H19, for example, has been shown to promote aerobic glycolysis through the miRNA/LDHA axis, thereby linking RNA regulatory networks to lactate production and malignant progression (38). Such findings emphasize that glycolytic reprogramming is sustained not only by canonical oncogenes or hypoxia-responsive pathways, but also by RNA-based circuitry that fine-tunes enzyme expression and metabolic output. These RNA-dependent mechanisms may be especially relevant in tumors where metabolic adaptation and immune escape evolve together under therapeutic or microenvironmental pressure. Relatedly, RNA stability regulators such as IGF2BP1 have emerged as key modulators of glycolysis-associated oncogenic signaling. By stabilizing transcripts such as c-Myc mRNA, IGF2BP1 can amplify glycolytic gene expression programs and enhance tumor metabolic plasticity (39). Notably, this type of post-transcriptional regulation also creates a mechanistic bridge between glycolysis and immune suppression, because c-Myc-centered metabolic activation can be accompanied by increased PD-L1 expression and reduced susceptibility to immune attack (40). These findings expand the conceptual framework of glycolytic regulation: rather than being controlled solely at the transcriptional or enzymatic level, glycolysis is embedded within multilayered oncogenic networks that include hypoxia signaling, growth control, epigenetic modulation, and RNA stability pathways. Together, these networks sustain the glycolytic phenotype while simultaneously predisposing tumors to immune evasion and therapy resistance.

### 2.3 Why glycolytic reprogramming has immunological consequences

The immunological significance of glycolytic reprogramming arises from the fact that tumor metabolism is inseparable from microenvironmental ecology. A highly glycolytic tumor does not simply consume more glucose for its own benefit; it actively reshapes the distribution of metabolic resources within the tumor microenvironment. Because glucose is also required for the activation, proliferation, and effector differentiation of immune cells, especially cytotoxic T lymphocytes, excessive tumor glucose consumption can impose a state of nutrient restriction on nearby immune populations (41). This creates a form of metabolic asymmetry in which tumor cells gain a competitive advantage while immune cells are pushed toward dysfunction, exhaustion, or reduced persistence.

**Figure 1:**
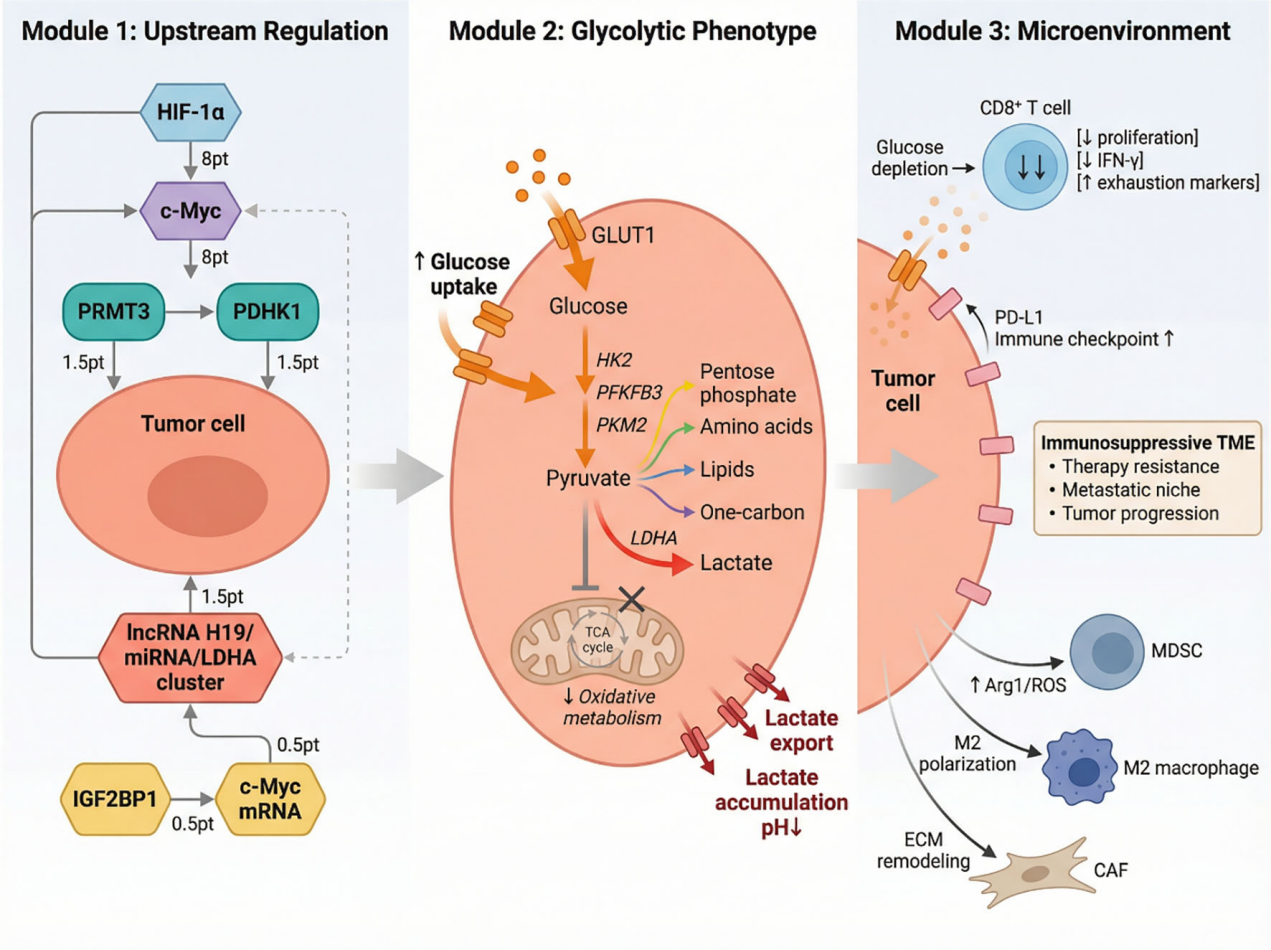
Molecular basis and immunological consequences of glycolytic reprogramming in cancer. Oncogenic and regulatory networks, including HIF-1α, c-Myc, PRMT3/PDHK1, H19-related RNA regulation, and IGF2BP1-mediated transcript stabilization, sustain glycolytic reprogramming in tumor cells. Enhanced glucose uptake, upregulation of key glycolytic enzymes, lactate accumulation, and reduced mitochondrial oxidation promote biosynthesis and stress adaptation while reshaping the tumor microenvironment through nutrient competition, acidification, PD-L1 upregulation, immune suppression, and therapy resistance.

At the same time, glycolytic reprogramming changes not only nutrient availability but also the composition of metabolic signals in the local environment. Increased lactate release and extracellular acidification create biochemical conditions that are unfavorable for effective antitumor immunity (2, 42, 43). These changes can impair T-cell proliferation, cytokine production, and cytotoxicity, while also influencing the differentiation and suppressive activity of myeloid cells and stromal populations. In this context, glycolysis-derived metabolites are not inert byproducts but active mediators of intercellular communication (44, 45). Their accumulation transforms the tumor microenvironment from a site of immune surveillance into a metabolically restrictive and immunoregulatory niche. This is why glycolytic reprogramming cannot be interpreted simply as a mechanism for enhanced ATP generation. Its broader consequence is the conversion of a tumor-intrinsic metabolic phenotype into a microenvironment-wide immunological state. Through nutrient depletion, lactate accumulation, acidification, and the activation of glycolysis-linked signaling pathways, cancer cells translate metabolic dominance into immune escape (46, 47). The result is a tumor ecosystem in which metabolic advantage and immunological suppression reinforce one another. Understanding this conversion is essential, because it explains how a metabolic program centered on glucose utilization can ultimately shape immune cell fate, checkpoint regulation, stromal cooperation, and treatment response. In other words, the immunological consequences of glycolytic reprogramming reflect a shift from cell-autonomous metabolism to ecosystem-level regulation. Once glycolysis is viewed through this broader lens, it becomes clear why metabolic traits that initially appear to support proliferation also carry direct implications for immune suppression and therapeutic resistance. This perspective provides the mechanistic basis for the subsequent sections of this review, which examine how glycolytic metabolism drives immune crosstalk, nutrient competition, and new therapeutic opportunities in cancer. **Figure 1** illustrates how oncogenic and post-transcriptional regulatory networks sustain the glycolytic phenotype in cancer cells and connect this metabolic state to microenvironmental remodeling. It also summarizes how glucose depletion, lactate accumulation, acidification, and PD-L1 upregulation translate tumor glycolysis into immune suppression and therapy resistance.

## 3 Glycolytic reprogramming drives immune crosstalk in the tumor microenvironment

### 3.1 Lactate as an immunoregulatory metabolite rather than a waste product

For many years, lactate was largely regarded as a passive end product of aerobic glycolysis and a biochemical indicator of metabolic inefficiency. This view is now clearly outdated. In tumors, lactate should be understood as an active metabolite with broad regulatory functions that extend well beyond intracellular redox maintenance (43, 48, 49). As glycolytic flux increases, the sustained conversion of pyruvate into lactate enables tumor cells to preserve NAD+ regeneration and maintain continuous glucose catabolism (50–52). However, the biological significance of this process lies not only in the support of tumor cell metabolism, but also in the fact that lactate is exported into the extracellular space, where it reshapes the tumor microenvironment and influences the behavior of multiple neighboring cell populations.

Rather than being metabolically inert, lactate acts as a signaling and microenvironment-modifying molecule that contributes to immune suppression at several levels. Its accumulation is commonly accompanied by extracellular acidification, altered nutrient gradients, and changes in cellular transport dynamics, all of which affect the function of immune and stromal cells. In this sense, lactate serves as a metabolic language through which highly glycolytic tumor cells communicate with surrounding cell types. It transmits the consequences of tumor-intrinsic metabolic reprogramming into the broader tissue ecosystem, thereby linking cancer cell metabolism to local immune dysfunction. This concept is particularly important in the context of tumor-associated myeloid cells and adaptive immune suppression. Studies in pancreatic cancer have shown that treatment-induced enhancement of glycolytic activity and lactate production can reinforce an immunosuppressive microenvironment, contributing to radioresistance through effects on myeloid-derived suppressor cells (MDSCs) (53–55). Similarly, work in breast cancer has demonstrated that tumor-derived lactate can regulate the inflammatory microenvironment by promoting macrophage polarization toward an M2-like state (56). Together, these findings indicate that lactate is not simply released as a metabolic byproduct but is actively involved in instructing immune composition and function within tumors. The significance of lactate extends beyond immune cells alone. Stromal elements, including CAF, can sense, import, and metabolically utilize lactate, thereby participating in reciprocal metabolic interactions with tumor cells (57, 58). Consequently, lactate occupies a central position in tumor immune crosstalk: it links glycolytic metabolism to immune suppression, stromal adaptation, and microenvironmental remodeling. Recognizing lactate as an immunoregulatory metabolite rather than a waste product is therefore essential for understanding how glycolytic tumors establish and maintain immune-evasive niches.

### 3.2 Lactate-mediated myeloid cell remodeling: MDSCs and TAMs

Among the immune populations most profoundly influenced by lactate are myeloid cells, which play central roles in establishing and maintaining tumor-associated immune suppression. Myeloid-derived suppressor cells (MDSCs) and tumor-associated macrophages (TAMs) are particularly sensitive to metabolic cues within the tumor microenvironment, and increasing evidence suggests that lactate is one of the key factors directing their suppressive phenotypes (59–63). This is highly relevant because myeloid cells often represent the dominant immunoregulatory population in solid tumors, where they can restrict T-cell function, support angiogenesis, remodel extracellular matrix, and promote resistance to therapy.

In the case of MDSCs, lactate appears to enhance their immunosuppressive activity and strengthen their contribution to therapeutic resistance. In pancreatic cancer, the study by Yang and colleagues provided a strong example of this principle by linking radiotherapy-induced metabolic adaptation to increased lactate production and enhanced MDSC-mediated immune suppression (64). This work is conceptually important because it shows that lactate-associated myeloid remodeling is not merely a baseline feature of tumor biology but can also emerge or intensify in response to treatment. In other words, therapy itself may amplify glycolytic immunosuppression, thereby creating a feed-forward loop that undermines therapeutic efficacy.

Macrophages are similarly shaped by lactate-rich environments. Rather than remaining in a pro-inflammatory, tumor-restrictive state, macrophages exposed to elevated lactate tend to acquire an M2-like phenotype characterized by tissue-remodeling, tumor-supportive, and immunosuppressive functions. The study by Wang and colleagues in breast cancer illustrates this process by showing that glycolysis-derived lactate can drive macrophage polarization and thereby regulate the inflammatory landscape of the tumor (65). This is a critical observation because it places lactate at the center of a phenotypic shift from antitumor immunity toward tumor tolerance. Through this mechanism, glycolytic tumors do not simply evade immune attack; they actively recruit and educate innate immune cells to support malignant progression.

The consequences of this lactate-mediated myeloid remodeling are multifaceted. MDSCs and M2-like TAMs suppress cytotoxic T-cell responses, produce immunosuppressive mediators, support vascular and stromal remodeling, and contribute to a tumor-permissive microenvironment that favors growth, invasion, and treatment resistance. Thus, lactate-driven myeloid reprogramming represents a crucial bridge between tumor metabolism and immune escape. It also highlights a broader principle: glycolytic metabolites can influence not only whether immune cells are present in tumors, but also what functional state they adopt once they arrive. This makes lactate a central organizer of innate immune remodeling in glycolysis-high tumors.

### 3.3 Fibroblast–tumor metabolic symbiosis and immunosuppressive niche formation

The impact of glycolytic reprogramming in tumors is not limited to tumor–immune interactions. It also extends to metabolic cooperation between tumor cells and stromal populations, particularly CAFs. Traditionally, CAFs were viewed mainly as structural and matrix-producing cells that contribute to desmoplasia and tissue stiffness. However, this interpretation is now too narrow. Increasing evidence indicates that CAFs are metabolically active participants in the tumor ecosystem and can engage in reciprocal nutrient exchange with cancer cells (66–69). This positions them as critical mediators of immune crosstalk and microenvironmental stabilization.

A particularly important example of this interaction is the reuse of tumor-derived lactate by CAFs. In pancreatic cancer, Kitamura and colleagues showed that CAFs can import and metabolize lactate released by glycolytic tumor cells, thereby maintaining a fibrotic and immunosuppressive microenvironment (70). This finding is highly significant because it expands the meaning of metabolic crosstalk from simple nutrient competition to metabolic symbiosis. Rather than functioning as passive bystanders, CAFs exploit tumor-derived metabolites to support their own persistence and to reinforce the pathological features of the microenvironment. This fibroblast–tumor metabolic cooperation has several important consequences. First, it helps sustain fibrosis and aberrant stromal architecture, both of which restrict perfusion and exacerbate hypoxia and nutrient heterogeneity. Second, it contributes to the maintenance of an immune-excluded or immune-impaired niche in which effector immune cells face both physical and metabolic barriers. Third, by stabilizing the stromal compartment, lactate-fueled CAF activity may indirectly protect tumor cells from therapeutic exposure and immune-mediated elimination. In this context, lactate does not merely suppress immunity through direct effects on immune cells; it also supports stromal programs that make the entire tumor ecosystem more resistant to immune infiltration and treatment.

These observations underscore an important conceptual shift. Immune crosstalk in glycolytic tumors should not be understood solely as communication between tumor cells and leukocytes. It also includes tumor–stroma metabolic cooperation that shapes immune accessibility, nutrient distribution, and suppressive niche formation. The CAF example highlights that glycolytic reprogramming creates a cooperative microenvironment in which stromal cells help perpetuate the consequences of tumor metabolism. This broader ecological view is essential for understanding why targeting cancer glycolysis may influence not only tumor growth but also stromal architecture and immune competence.

### 3.4 Glycolysis-linked regulation of immune checkpoints

One of the most important advances in recent years has been the recognition that glycolytic reprogramming can directly regulate immune checkpoint expression. This concept substantially broadens the traditional model of checkpoint induction, which has often emphasized inflammatory cytokines, oncogenic signaling, or adaptive immune feedback as the dominant drivers of PD-L1 expression. While those pathways remain important, it is now evident that metabolic programs themselves can actively shape checkpoint regulation. In other words, PD-L1 expression is not always simply a downstream consequence of inflammation; in some tumors, it can be built into the glycolytic state itself (71–74). A landmark example of this principle comes from the study by Guo and colleagues, who demonstrated that aerobic glycolysis promotes tumor immune evasion through HK2-mediated phosphorylation of IκBα (75). Mechanistically, this work showed that under glucose-rich conditions, HK2 can disengage from its classical mitochondrial localization and participate in signaling events that ultimately promote PD-L1 expression. This finding is particularly influential because it provides a direct mechanistic bridge between glucose metabolism and checkpoint control. It shows that glycolytic enzymes are not merely metabolic catalysts but can also function as signaling regulators that translate nutrient conditions into immune-evasive outputs.

A second important axis is represented by PRMT3/PDHK1-dependent regulation of glycolysis. In hepatocellular carcinoma, PRMT3 was shown to activate PDHK1-regulated glycolysis and thereby drive PD-L1-mediated immune escape (37). This study is notable because it connects an upstream regulatory factor with metabolic rerouting and a defined immune checkpoint outcome. By diverting pyruvate away from mitochondrial oxidation and reinforcing lactate-producing glycolysis, the PRMT3/PDHK1 pathway supports a metabolic state that is not only growth-promoting but also immunosuppressive. This reinforces the idea that signaling networks sustaining glycolysis may also serve as checkpoint-regulatory programs. Post-transcriptional regulation adds another important layer to this mechanism. Recent work on IGF2BP1 has shown that RNA stability control can couple c-Myc activation, glycolytic enhancement, and immune escape in hepatocellular carcinoma (39). By stabilizing c-Myc transcripts, IGF2BP1 amplifies glycolytic gene expression and promotes a metabolic phenotype associated with increased PD-L1 expression and reduced antitumor immune susceptibility. This is conceptually valuable because it demonstrates that checkpoint regulation can emerge not only from direct signaling cascades but also from RNA-centered control of metabolic oncogenes. It further suggests that immune escape may be embedded within broader post-transcriptional programs of metabolic adaptation.

**Figure 2:**
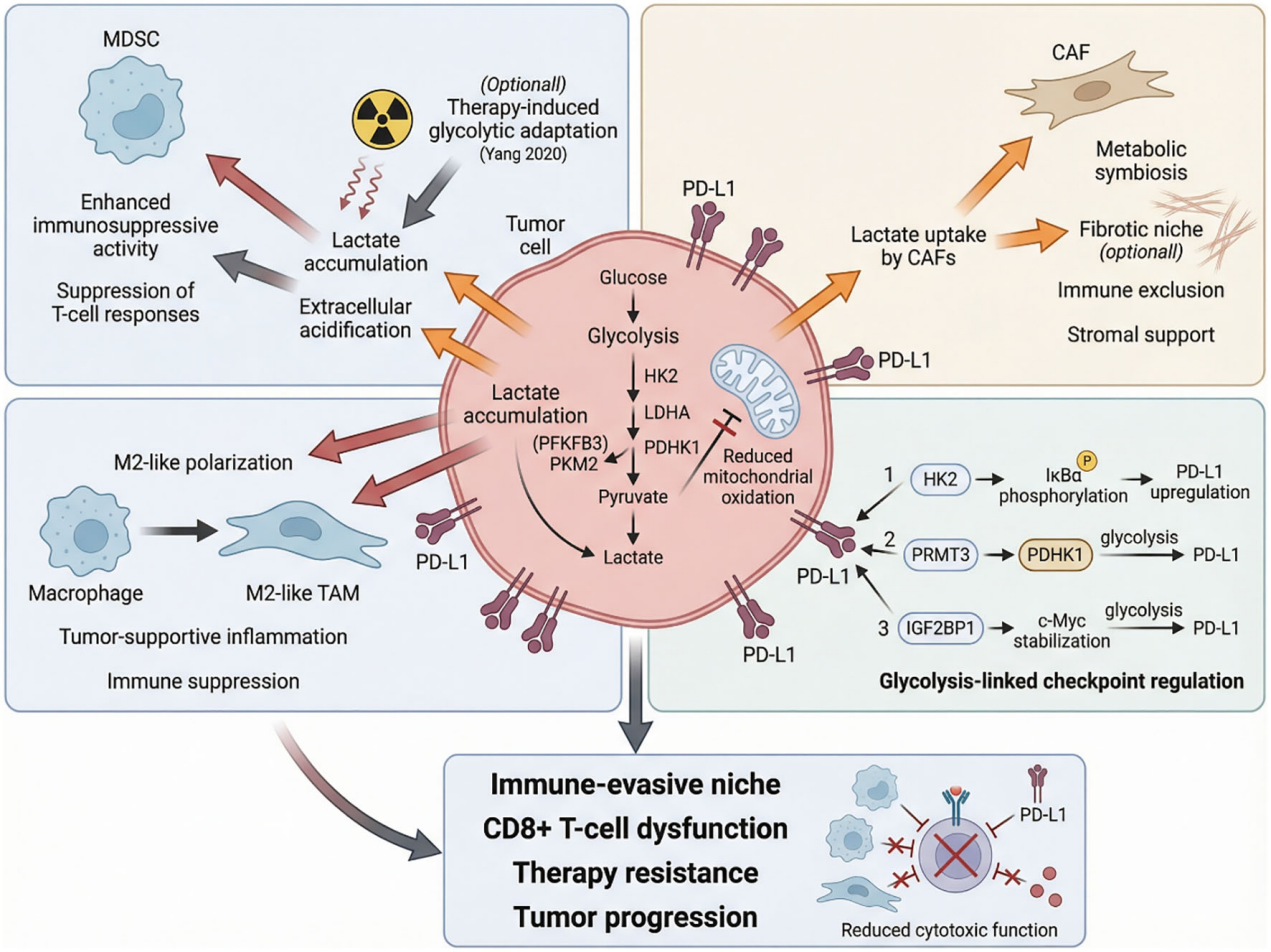
Lactate-centered immune crosstalk driven by glycolytic reprogramming in the tumor microenvironment. Enhanced tumor glycolysis increases lactate production, extracellular acidification, and PD-L1-associated immune escape. Lactate acts as an active immunoregulatory metabolite by promoting MDSC suppression, M2-like macrophage polarization, and CAF-mediated stromal support, while glycolysis-linked signaling pathways directly reinforce checkpoint regulation. Together, these processes establish an immune-evasive and therapy-resistant tumor niche.

Taken together, the HK2/IκBα/PD-L1, PRMT3/PDHK1/glycolysis/PD-L1, and IGF2BP1/c-Myc/glycolysis/PD-L1 axes illustrate a common principle: glycolytic reprogramming can directly instruct immune checkpoint expression through multiple mechanistic routes. These routes include metabolic enzyme signaling, mitochondrial metabolic diversion, and RNA stability-mediated amplification of oncogenic metabolism. This convergence is highly important for the overall logic of the present review. It indicates that immune checkpoints are not simply immunological markers superimposed on tumor cells, but can be integral outputs of tumor metabolic architecture. Such a view helps explain why glycolysis-high tumors often display pronounced immune suppression and why combining metabolic targeting with checkpoint blockade may be a particularly rational therapeutic strategy. To clarify the experimental basis of these mechanisms, we further summarized the model systems used in representative studies, including tumor cell–immune cell co-culture assays, mouse tumor models, radiotherapy-treated preclinical models, engineered T-cell systems, and analyses of human tumor tissues or patient-derived data where available (**Table 1**). This distinction is important because some mechanisms are supported mainly by preclinical models, whereas others have additional human correlative evidence. **Figure 2** summarizes how lactate functions as a central mediator linking tumor glycolysis to immune and stromal remodeling in the tumor microenvironment. It also highlights that glycolysis-driven immune escape is reinforced not only by lactate-dependent myeloid and CAF reprogramming, but also by direct upregulation of PD-L1 through metabolism-linked signaling pathways.

**Table 1:** Experimental models and human relevance of representative studies on glycolysis-mediated immune regulation in cancer.

Mechanistic focus	Representative study	Cancer type/disease context	Experimental model used	Human relevance	Main finding	DOI
Lactic acid–PD-1 axis in Tregs	Kumagai et al., *Cancer Cell*, 2022	Highly glycolytic tumor microenvironment	Mechanistic analysis of regulatory T cells in glycolytic tumor settings; lactate/MCT1-related functional assays	Directly relevant to tumor immune suppression; study links glycolytic tumors with PD-1 induction in Tregs	Lactic acid promoted PD-1 expression in Tregs through MCT1-dependent uptake, suggesting that lactate functions as a metabolic immune checkpoint in glycolysis-high tumors.	10.1016/j.ccell.2022.01.001
Lactate-mediated MDSC immunosuppression and radioresistance	Yang et al., *Cancer Immunology Research*, 2020	Pancreatic cancer	Preclinical pancreatic cancer radiotherapy models and MDSC-focused immune assays	Mainly preclinical; relevant to radiotherapy-associated immune suppression in pancreatic cancer	Radiation-enhanced glycolysis increased lactate secretion, which promoted MDSC-mediated immunosuppression and contributed to pancreatic cancer radioresistance.	10.1158/2326-6066.CIR-20-0111
Tumor-derived lactate and macrophage polarization	Mu et al., *Cell Cycle*, 2018	Breast cancer	Breast cancer cell–macrophage experimental systems; mechanistic pathway analysis	Preclinical evidence; relevant to macrophage polarization in breast cancer TME	Tumor-derived lactate induced M2-like macrophage polarization through ERK/STAT3 signaling, supporting lactate as an active regulator of TAM phenotype.	10.1080/15384101.2018.1444305
CAF lactate utilization and immunosuppressive fibrosis	Kitamura et al., *JCI Insight*, 2023	Pancreatic ductal adenocarcinoma	CAF-rich murine PDAC model; cancer cell–CAF metabolic interaction analysis; LDHA-related tumor lactate assessment	Includes human relevance because LDHA expression was associated with poor prognosis in patients with PDAC	CAFs under glucose-restricted conditions reused cancer-derived lactate as a metabolic fuel, maintaining fibrotic and immunosuppressive PDAC microenvironments.	10.1177/17456916231188052
HK2-mediated glycolysis and PD-L1 upregulation	Guo et al., *Cell Metabolism*, 2022	Solid tumor immune evasion	Tumor cell mechanistic assays and immune checkpoint blockade combination models	Translationally relevant because HK inhibition enhanced anti-PD-1 efficacy in preclinical treatment settings	Aerobic glycolysis promoted tumor immune evasion through HK2-mediated phosphorylation of IκBα, leading to NF-κB activation and PD-L1 upregulation.	10.1016/j.cmet.2022.08.002
PRMT3/PDHK1/glycolysis/PD-L1 axis	Ding et al., *Cell Death & Disease*, 2025	Hepatocellular carcinoma	Hepatocyte-specific Prmt3 knockout in DEN-CCL4-induced HCC mice; HCC cell assays; RNA-seq; ChIP; tissue microarray	Strong human relevance because tissue microarray showed positive correlation between PRMT3 and PD-L1 in HCC patients	PRMT3 activated PDHK1-regulated glycolysis, increased lactate production, enhanced H3K18 lactylation at the PD-L1 promoter, and promoted PD-L1-mediated immune escape.	10.1038/s41419-025-07482-7
IGF2BP1/c-Myc/glycolysis/PD-L1 axis	Ye et al., *Frontiers in Immunology*, 2024	Hepatocellular carcinoma	HCC cell glycolysis assays; CD8+ T cell-mediated killing assays; luciferase, immunoprecipitation, and ChIP-PCR analyses	Human relevance supported by association of elevated IGF2BP1 with poor prognosis and reduced CD8+ T-cell infiltration in HCC patients	IGF2BP1 stabilized c-Myc mRNA, enhanced aerobic glycolysis, increased PD-L1 expression, suppressed CD8+ T-cell cytotoxicity, and promoted oxaliplatin resistance.	10.3389/fimmu.2024.1480834
Tumor glycolysis and T-cell immune surveillance	Liu et al., *Cell Metabolism*, 2021	Solid tumors	Tumor cell metabolic and immune surveillance models	Mainly preclinical, with broad relevance to immune evasion mechanisms	Tumors exploited FTO-mediated regulation of glycolytic metabolism to evade immune surveillance, supporting the link between tumor glycolysis and impaired antitumor immunity.	10.1016/j.cmet.2021.04.001
GLUT1-overexpressing CAR-T cells	Guerrero et al., *Nature Communications*, 2024	CAR-T cell therapy	Primary human CAR-T cells; *in vitro* functional assays; *in vivo* tumor challenge models	High translational relevance because the engineered cells were primary human CAR-T cells	GLUT1 overexpression increased glucose consumption, glycolysis, glycolytic reserve, oxidative phosphorylation, cytokine secretion, tumor killing, and *in vivo* CAR-T persistence.	10.1038/s41467-024-52666-y
GLUT3-overexpressing CAR-T cells	Hu et al., *Journal for ImmunoTherapy of Cancer*, 2025	Solid tumor CAR-T therapy	CAR-T functional assays under glucose restriction; xenograft and syngeneic mouse tumor models	Translational/preclinical relevance for low-glucose solid tumors	Glut3 overexpression enhanced glucose uptake, mitochondrial fitness, cytokine production, and antitumor efficacy of CAR-T cells under metabolically restrictive conditions.	10.1136/jitc-2024-010540
HK2-engineered T cells	Zur et al., *Frontiers in Immunology*, 2025	Engineered T-cell therapy	Primary human T cells engineered to overexpress HK2 with tumor-specific receptor; *in vitro* and human tumor xenograft models	Translational/preclinical relevance for adoptive T-cell therapy	HK2-engineered T cells showed increased glycolytic capacity, cytokine secretion, activation marker expression, delayed tumor growth, and improved survival *in vivo*.	10.3389/fimmu.2025.1477929
Genetic enhancement of glycolysis in T cells	Zur et al., *Journal for ImmunoTherapy of Cancer*, 2024	Adoptive T-cell therapy	Human T cells engineered to express glycolysis-related components together with tumor-specific CAR or TCR	Translational/preclinical relevance	Enhancing glycolytic capacity improved T-cell antitumor function and helped immune cells better compete with tumor cells for metabolic resources.	10.1136/jitc-2023-008434

### 3.5 ILC2s and tissue-resident immune responses in glycolytic tumor niches

Although T cells, myeloid cells, CAFs, and immune checkpoints represent major components of glycolysis-driven immune escape, this framework should be expanded to include tissue-resident immune populations (76–78). Group 2 innate lymphoid cells (ILC2s) are increasingly recognized as regulators of type 2 immunity, tissue repair, stromal remodeling, and tumor–immune interactions (79, 80). In melanoma models, Wagner et al. showed that the IL-33–ILC2–eosinophil axis can support antitumor immunity, whereas tumor-derived lactic acid limits this response and contributes to impaired ILC2-associated immune activity (81). This study directly links tumor lactate accumulation to tissue-resident type 2 immune regulation and provides an important example of how glycolytic metabolites may reshape innate antitumor immunity.

Beyond lactate, nutrient deprivation and stromal-derived signals may also influence ILC2 behavior in tumors (82). Glycolysis-high tumor niches are often characterized by glucose depletion, acidosis, hypoxia, and CAF-rich stromal remodeling (83, 84). These conditions may interact with epithelial- and stromal-derived alarmins such as IL-33, IL-25, and thymic stromal lymphopoietin, which are well-known regulators of ILC2 activation and expansion (80, 85). ILC2s are themselves metabolically responsive cells, and their cytokine production, survival, and tissue-repair functions may be shaped by nutrient availability and local metabolic stress. Marciniak et al. further highlighted tumor-derived lactic acid as a potential regulator of innate lymphoid cells and emphasized its relevance in barrier-tissue tumors, where tissue-resident immune responses are tightly linked to metabolic and stromal cues (86). Therefore, ILC2s may represent an underexplored link between glycolytic reprogramming, type 2 immune regulation, tissue repair-like inflammation, and stromal remodeling. Future studies should determine whether lactate, nutrient deprivation, and CAF-derived signals directly regulate ILC2 localization, cytokine programs, checkpoint expression, and functional cooperation with myeloid or stromal cells in glycolysis-high tumor niches.

## 4 Nutrient competition and metabolic stress in antitumor immunity

### 4.1 Tumor cells outcompete immune cells for glucose

Nutrient competition is one of the most direct yet often underappreciated mechanisms by which tumors suppress antitumor immunity. In glycolysis-high cancers, tumor cells avidly consume glucose and establish a state of local nutrient depletion within the tumor microenvironment. Because glucose is not only a fuel for malignant proliferation but also a critical substrate for immune activation, this metabolic asymmetry creates a profound disadvantage for antitumor immune cells. In effect, tumor cells do not merely use glucose to support their own growth; they also deprive neighboring immune populations of a resource that is essential for their effector function (87, 88).

This issue is particularly important for CD8+ T cells. Once activated, cytotoxic T lymphocytes undergo marked metabolic reprogramming and rely on increased glucose uptake to sustain clonal expansion, cytokine production, biosynthetic activity, and cytotoxic killing. Their antitumor activity is therefore tightly linked to metabolic sufficiency. In glucose-restricted environments, however, T cells face a dual challenge: limited substrate availability constrains energy production, while reduced glycolytic flux also compromises the generation of metabolic intermediates required for activation and effector differentiation (89–92). As a result, immune dysfunction in tumors is not simply a matter of inhibitory signaling but also one of metabolic insufficiency. This perspective helps explain why T cells can remain present in tumors yet fail to exert durable control over cancer growth. When tumor cells dominate glucose utilization, they impose a competitive metabolic hierarchy in which immune cells are forced to operate below the threshold needed for optimal function. Under these conditions, T cells may exhibit impaired proliferation, reduced cytokine secretion, diminished cytotoxicity, and a greater tendency toward dysfunctional or exhausted states. Thus, nutrient competition should be viewed not as a secondary consequence of tumor growth, but as a central mechanism through which glycolytic tumors actively undermine immune surveillance. More broadly, glucose competition illustrates a key ecological principle of the tumor microenvironment: cells with the most aggressive metabolic program can shape the functional capacity of surrounding cell populations. In highly glycolytic tumors, metabolic advantage becomes immunological advantage. By monopolizing glucose, tumor cells create a bioenergetic bottleneck for antitumor lymphocytes, thereby translating metabolic dominance into immune escape.

### 4.2 Lactic acid and acidic stress impair T-cell metabolic fitness

The metabolic burden imposed on T cells in tumors is not limited to nutrient deprivation alone. Glycolysis-high tumors also generate large amounts of lactate and acidify the extracellular environment, creating a second layer of metabolic stress that further compromises immune function. This is a critical point, because nutrient competition in cancer is not simply a matter of tumor cells “consuming too much”; it also involves the accumulation of harmful metabolic byproducts that actively suppress immune competence.

Lactate exerts inhibitory effects on T-cell function through multiple mechanisms (93–96). Elevated extracellular lactate can interfere with cellular metabolic balance, limit effective glycolytic activity, and disrupt the ability of T cells to maintain the bioenergetic and biosynthetic demands required for effector responses. At the same time, extracellular acidification imposes an unfavorable biochemical environment that impairs proliferation, cytokine secretion, and cytotoxic activity. Thus, T cells in tumors face both a lack of essential nutrients and continuous exposure to a metabolically hostile milieu. This combination is particularly detrimental because activated T cells themselves depend on dynamic metabolic reprogramming. Their transition from quiescence to effector activity requires not only receptor signaling but also the ability to sustain glucose uptake and flux through glycolytic pathways. When lactate accumulates and pH declines, this metabolic adaptation becomes increasingly difficult to maintain. T cells may therefore lose functional fitness even when they are antigen-experienced or physically present within the tumor. In this context, lactate acts not merely as a marker of glycolytic metabolism but as an active barrier to effective antitumor immunity.

Importantly, this means that nutrient competition should be conceptualized in broader terms than simple resource scarcity. The tumor microenvironment is shaped by a combination of glucose deprivation and metabolite toxicity. Together, these forces create a landscape in which effector lymphocytes are weakened both by what is missing and by what has accumulated. This dual pressure helps explain why T-cell dysfunction is often so persistent in glycolysis-high tumors and why reversing immune suppression may require more than checkpoint blockade alone.

### 4.3 Glucose transport and metabolic resilience in CD8+ T cells

If glucose competition is a major determinant of antitumor immune failure, then the capacity of T cells to acquire and utilize glucose becomes a critical variable in therapeutic success. Recent evidence suggests that antitumor CD8+ T cells are not intrinsically equipped to withstand severe metabolic restriction and that specific glucose transport mechanisms are required to preserve their activation and function in the tumor microenvironment (97–100). This has shifted attention from tumor-centered metabolism alone to the question of how immune cells maintain metabolic resilience under competitive stress. A notable example is the role of GLUT10 in CD8+ T-cell biology. Recent work has shown that GLUT10 supports T-cell activation and antitumor immunity, highlighting the importance of glucose transport capacity in sustaining effective immune responses (101). This finding is conceptually important because it argues against the assumption that activated T cells can automatically adapt to low-glucose conditions. Instead, their functional persistence depends on maintaining adequate access to metabolic substrates, and disruption of this process can directly impair antitumor activity.

The importance of glucose transport also reframes how we think about T-cell dysfunction in tumors. T-cell failure may not only reflect excessive inhibitory signaling or terminal exhaustion, but also an inability to secure sufficient metabolic input for continued function. In this model, glucose transporters act as gatekeepers of immune competence, determining whether CD8+ T cells can maintain the metabolic programs necessary for proliferation, effector molecule production, and cytotoxic killing. The tumor microenvironment therefore becomes not only an immunosuppressive niche but also a test of metabolic adaptability. Additional studies further support the broader idea that preserving metabolic fitness in T cells can improve antitumor immunity. Work implicating regulators such as HIF-1α-associated pathways in cytotoxic T-cell fitness suggests that the metabolic state of T cells is itself an actionable determinant of therapeutic outcome (102). Taken together, these findings indicate that effective antitumor immunity depends not simply on the presence of CD8+ T cells, but on whether they retain the metabolic infrastructure required to respond under conditions of glucose limitation and metabolic stress.

### 4.4 Metabolic engineering of T cells to overcome glucose restriction

One of the most exciting developments in this field is the emergence of strategies that directly engineer T cells to improve their metabolic competitiveness in the tumor microenvironment. These approaches are based on a straightforward but powerful principle: if tumors suppress immunity in part by dominating glucose utilization, then enhancing the glycolytic capacity of therapeutic T cells may help restore functional balance. This idea moves the field beyond describing metabolic suppression toward actively redesigning immune cells to resist it.

Several recent studies provide compelling support for this concept. Engineering T cells to enhance glycolysis has been shown to improve antitumor function, indicating that metabolic reinforcement can directly increase immune fitness in the face of tumor-associated nutrient restriction (103). In adoptive cell therapy models, overexpression of GLUT1 in CAR-T cells induces broader metabolic reprogramming and enhances functional potency (104). This suggests that improving glucose transport can reinforce not only substrate uptake but also the downstream metabolic architecture needed for sustained antitumor activity. Similarly, overexpression of GLUT3 has been shown to improve glucose uptake and antitumor efficacy under environmentally restrictive conditions (105). This is particularly relevant for solid tumors, where glucose availability is often low and metabolically hostile conditions are common. By increasing access to limited glucose pools, engineered T cells may better maintain proliferation, persistence, and cytotoxic function despite competition from highly glycolytic tumor cells. These findings provide a direct translational answer to one of the central problems outlined earlier in this review: T cells do not inevitably fail in low-glucose tumors if their metabolic fitness can be deliberately enhanced. Further strengthening this concept, HK2-engineered T cells have been reported to display increased antitumor responses and to synergize with anti-PD-1 immunotherapy (106). This is especially important because it connects metabolic engineering with checkpoint-based therapy rather than treating them as separate treatment paradigms. It suggests that metabolic enhancement can increase the capacity of T cells to respond once inhibitory signaling is relieved, thereby providing a rational basis for combination strategies. In such a framework, checkpoint blockade may remove suppressive brakes, whereas metabolic engineering equips T cells with the bioenergetic capacity needed to exploit that opportunity.

**Figure 3:**
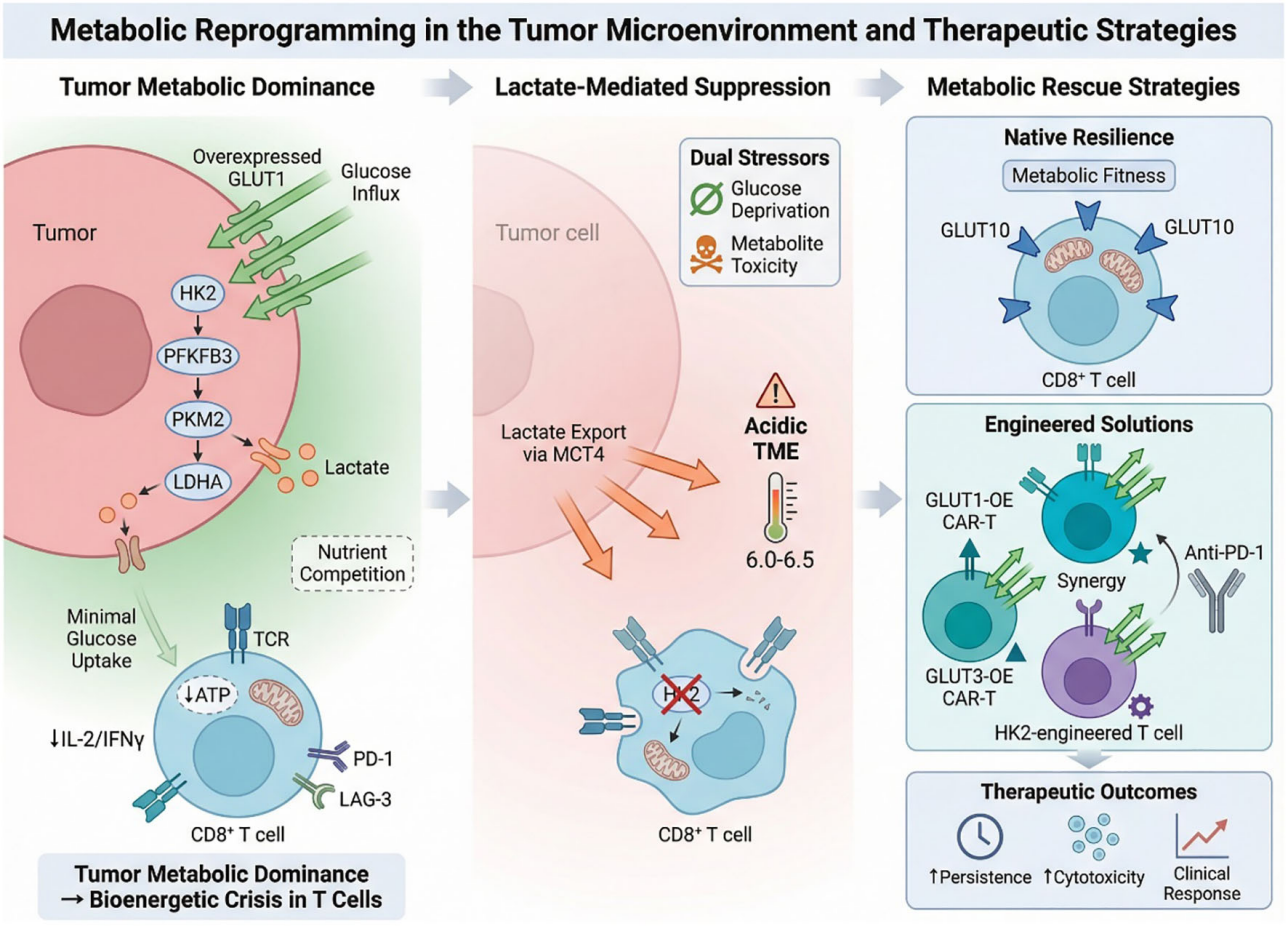
Nutrient competition and metabolic stress impair antitumor T-cell function in glycolysis-high tumors. Highly glycolytic tumor cells deplete glucose and accumulate lactate, creating a metabolically hostile microenvironment that restricts CD8+ T-cell activation, proliferation, and cytotoxicity. Glucose transport capacity, including GLUT10, supports T-cell fitness, whereas engineered T cells with enhanced GLUT1, GLUT3, or HK2 activity may better withstand low-glucose conditions and improve responses to immunotherapy.

Collectively, these studies point to a major shift in the therapeutic logic of cancer immunometabolism. Instead of focusing exclusively on suppressing tumor glycolysis, it may be equally important to increase the metabolic adaptability of immune cells themselves. Enhancing glucose transport and glycolytic capacity in T cells does not simply make them more active *in vitro*; it directly addresses the ecological constraints imposed by the tumor microenvironment. This makes metabolic engineering a highly promising strategy for overcoming nutrient competition and improving the efficacy of immunotherapy, particularly in metabolically restrictive solid tumors. Key studies showing how nutrient competition and T-cell metabolic adaptation shape antitumor immunity are summarized in **Table 2**. **Figure 3** summarizes how tumor glycolysis imposes dual metabolic pressure on CD8+ T cells through glucose deprivation and lactate-associated acidic stress. It also highlights that enhancing glucose transport and glycolytic capacity in therapeutic T cells may help overcome nutrient competition and improve antitumor efficacy.

**Table 2:** Representative studies on nutrient competition, T-cell metabolic fitness, and metabolic engineering strategies.

Category	Study	Model/cell type	Main finding	Relevance to this review	DOI
Glucose transport in CD8+ T cells	Liu Y, et al. *Sci Transl Med.* 2024	CD8+ T cells/tumor models	GLUT10 supported CD8+ T-cell activation and antitumor immunity, whereas lactic acid disrupted this process	Directly supports the concept that nutrient competition and lactate impair T-cell function	10.1126/scitranslmed.adk7399
Engineering glycolysis in T cells	Toledano Zur R, et al. *J Immunother Cancer.* 2024	Engineered T cells	Genetic enhancement of glycolysis increased T-cell antitumor function	Provides proof-of-concept that improving T-cell glycolysis can overcome metabolic restriction	10.1136/jitc-2023-008434
GLUT1-based CAR-T metabolic enhancement	Guerrero JA, et al. *Nat Commun.* 2024	CAR-T cells	GLUT1 overexpression induced metabolic reprogramming and enhanced CAR-T potency	Supports transporter-based engineering to improve immune-cell competitiveness	10.1038/s41467-024-52666-y
GLUT3-based adaptation to low-glucose TME	Hu W, et al., 2025	CAR-T cells/restrictive microenvironment	Glut3 overexpression improved environmental glucose uptake and antitumor efficacy	Reinforces the importance of glucose acquisition in metabolically hostile tumors	10.1136/jitc-2024-010540
HK2-engineered T cells	Zur RT, et al., 2025	Engineered T cells	HK2-engineered T cells showed stronger antitumor responses and synergized with anti-PD-1 therapy	Highlights the translational value of combining metabolic engineering with immunotherapy	10.3389/fimmu.2025.1477929
T-cell metabolic fitness and HIF-1α-related control	Liu J, et al. *J Immunother Cancer.* 2025	Cytotoxic T cells/tumor models	GP73 reinforced cytotoxic T-cell function by regulating HIF-1α and improved antitumor efficacy	Supports the broader concept of restoring T-cell metabolic fitness in cancer therapy	10.1136/jitc-2024-009265

## 5 Glycolytic control of immune escape and therapy resistance

### 5.1 From metabolic adaptation to immune escape

The evidence discussed above supports a central conclusion: glycolytic reprogramming is not merely a metabolic adaptation that allows tumor cells to sustain proliferation under stress, but a multifaceted driver of immune escape. Its immunological impact emerges through several converging mechanisms that together reshape the tumor microenvironment into a state that is unfavorable for effective antitumor immunity. In this way, a metabolic phenotype that initially appears tumor-intrinsic becomes translated into a broader ecosystem-level immunosuppressive program.

One major route through which glycolysis promotes immune escape is the regulation of immune checkpoint expression. As described in previous sections, glycolysis-associated pathways can directly enhance PD-L1 expression through distinct molecular mechanisms, including the HK2/IκBα axis, the PRMT3/PDHK1 pathway, and IGF2BP1/c-Myc-mediated metabolic activation. These findings are important because they show that checkpoint upregulation is not always simply a consequence of inflammatory signaling or adaptive immune pressure. Instead, tumor cells can integrate metabolic rewiring into immune checkpoint control, thereby embedding immune evasion within the architecture of their metabolic state.

At the same time, glycolytic metabolism shapes immune escape indirectly through the accumulation of lactate and the generation of an acidic extracellular milieu. Lactate-rich conditions promote the suppressive activity of MDSCs and favor macrophage polarization toward tumor-supportive M2-like states, thereby weakening cytotoxic immunity and reinforcing immunoregulatory myeloid networks (107–111). In parallel, lactate and acidification impair T-cell proliferation, cytokine production, and cytotoxic function, reducing the capacity of effector lymphocytes to restrain tumor progression. Thus, lactate serves as both a metabolic consequence of glycolysis and an active mediator of immune suppression. The role of stromal cells further expands this framework. CAFs can recycle tumor-derived lactate and contribute to the maintenance of fibrotic, poorly perfused, and immunosuppressive niches. This fibroblast–tumor metabolic cooperation strengthens immune exclusion and supports a microenvironment in which immune infiltration and function are both compromised (112, 113). Therefore, glycolytic immune escape is not restricted to direct tumor–immune interactions, but also involves stromal programs that stabilize and perpetuate the suppressive microenvironment. Finally, glycolysis-driven nutrient competition imposes a critical energetic constraint on antitumor immunity. By consuming glucose at high rates, tumor cells deprive CD8+ T cells of a substrate required for activation, expansion, and effector activity. The resulting metabolic stress reduces T-cell fitness precisely where immune function is most needed. Taken together, PD-L1 upregulation, lactate-driven myeloid remodeling, fibroblast-supported immune exclusion, glucose competition, and acid-mediated T-cell dysfunction form an interconnected network through which glycolytic reprogramming drives immune escape. Rather than acting through a single pathway, tumor glycolysis orchestrates immune suppression across multiple cellular and molecular levels.

### 5.2 Glycolysis and resistance to radiotherapy, chemotherapy, and immunotherapy

The same glycolytic mechanisms that support immune escape also contribute directly to resistance against major anticancer therapies. This is not surprising, because therapy resistance and immune suppression are often biologically intertwined. Tumors that survive treatment frequently do so not only by activating intrinsic stress-response pathways, but also by reshaping the surrounding microenvironment in ways that limit immune-mediated clearance. Glycolytic reprogramming occupies a central position in this process.

In the context of radiotherapy, glycolysis can support resistance by fostering an immunosuppressive post-treatment microenvironment (114–117). The study by Yang and colleagues in pancreatic cancer is particularly informative in this regard. Their work showed that radiotherapy can be accompanied by enhanced glycolytic activity and increased lactate production, which in turn promote MDSC-mediated immunosuppression and contribute to radioresistance. This finding illustrates an important principle: treatment itself can intensify the very metabolic conditions that undermine its effectiveness. Rather than simply eliminating tumor cells, radiotherapy may also select for or amplify glycolytic programs that support immune suppression and residual disease survival.

Glycolysis is also increasingly implicated in chemotherapy resistance and broader drug tolerance (118–121). In hepatocellular carcinoma, the IGF2BP1/c-Myc axis provides a clear example of how post-transcriptional enhancement of glycolysis can be linked to both immune escape and oxaliplatin resistance. This suggests that chemotherapy resistance is not solely determined by drug transport, DNA repair, or apoptosis pathways, but may also arise from metabolic programs that reinforce tumor adaptability while simultaneously reducing immune vulnerability. Related therapeutic studies that suppress glycolysis in colorectal cancer further support this view by showing that interfering with tumor glycolysis can reduce adaptive immune resistance and improve treatment responsiveness. These findings place glycolysis at the intersection of tumor cell survival and therapy-associated immune remodeling. The implications for immunotherapy are equally significant. Glycolysis-high tumors are particularly prone to developing resistance to checkpoint blockade because they combine several unfavorable features: elevated PD-L1 expression, reduced T-cell metabolic fitness, lactate-driven myeloid suppression, and persistent microenvironmental stress (122–124). Even when inhibitory receptor signaling is targeted therapeutically, immune cells may remain metabolically incapable of mounting an effective response. In this setting, adaptive immune tolerance is sustained not only by canonical checkpoint pathways but also by a persistent ecological imbalance in nutrients and metabolites. This helps explain why checkpoint inhibition alone often produces incomplete or transient benefit in metabolically hostile tumors. Collectively, these observations indicate that glycolysis contributes to resistance across multiple treatment modalities, including radiotherapy, chemotherapy, and immunotherapy. Its role is not confined to tumor cell bioenergetics; rather, it shapes the treatment response by reorganizing immune composition, stromal support, checkpoint expression, and metabolic accessibility within the tumor niche. Glycolytic reprogramming therefore emerges as a shared mechanism through which tumors endure therapeutic stress while maintaining immune escape.

### 5.3 Glycolysis as a convergence point between tumor-intrinsic and microenvironmental resistance

A particularly important feature of glycolytic reprogramming is that it links tumor-intrinsic resistance mechanisms with microenvironmental adaptation. These two dimensions of resistance are often discussed separately: one centered on the cancer cell’s own survival machinery, and the other on the suppressive properties of the surrounding tumor niche. However, glycolysis demonstrates that these dimensions are deeply interconnected. It is simultaneously a cell-autonomous survival program and a microenvironmental organizing force. From the tumor-intrinsic perspective, glycolysis supports proliferation, biosynthesis, redox balance, and adaptation to hypoxia or therapeutic stress. It allows cancer cells to maintain metabolic flexibility under conditions in which mitochondrial oxidation may be limited or unstable. Through the activity of enzymes such as HK2, LDHA, and PDHK1, as well as upstream regulators such as HIF-1α, c-Myc, and RNA stability factors, tumor cells build a metabolic program that sustains growth and protects against stress-induced collapse. These features alone make glycolysis a key determinant of malignant persistence.

**Figure 4:**
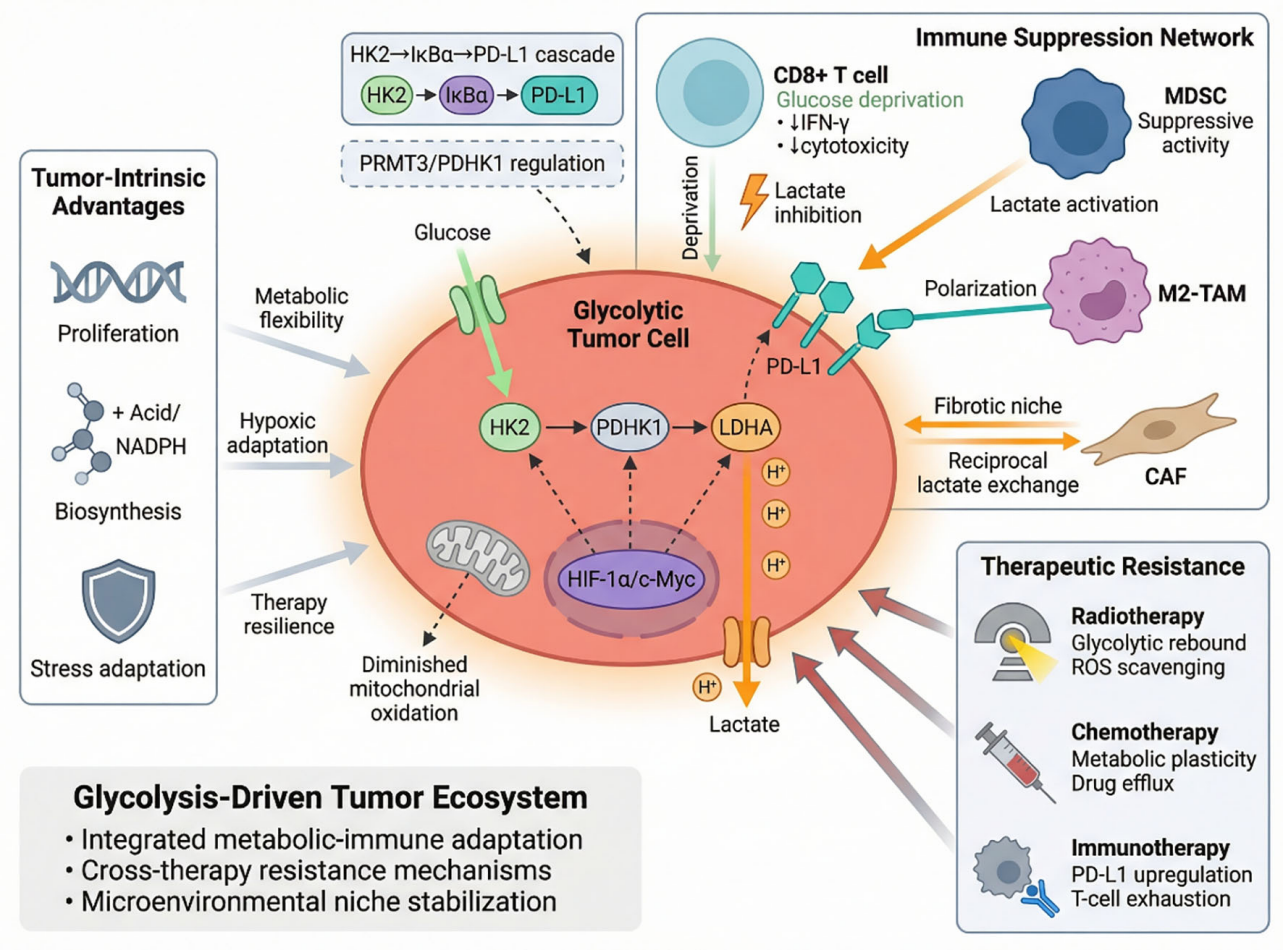
Glycolytic reprogramming acts as a convergence hub linking immune escape to therapy resistance in cancer. Enhanced tumor glycolysis supports proliferation and stress adaptation while promoting PD-L1 expression, lactate-driven myeloid suppression, CAF-mediated immune exclusion, glucose competition, and T-cell dysfunction. These coordinated effects reshape the tumor microenvironment into an immune-evasive niche and contribute to resistance to radiotherapy, chemotherapy, and immunotherapy.

Yet the significance of glycolysis extends far beyond intracellular survival. By depleting glucose, releasing lactate, acidifying the extracellular space, and shaping stromal cooperation, glycolytic tumors reorganize the surrounding microenvironment into an immunosuppressive and therapy-resistant niche. In this setting, resistance is no longer only a property of the tumor cell itself; it becomes a distributed characteristic of the entire tumor ecosystem. Immune cells lose function, myeloid populations become suppressive, fibroblasts reinforce exclusionary architecture, and therapeutic responses are blunted. Thus, glycolysis serves as a bridge between what tumor cells do internally and what they impose externally on the tissue environment. This convergence is highly relevant for translational strategy. Because glycolysis connects tumor survival with microenvironmental suppression, it represents a particularly attractive target for combination therapy. Interventions aimed at glycolysis may not only weaken tumor metabolic fitness but also restore immune function, reduce checkpoint-associated resistance, and disrupt stromal support. In other words, targeting glycolysis has the potential to attack both sides of the resistance equation at once: the intrinsic resilience of malignant cells and the extrinsic suppressive structure of the tumor microenvironment (125–128). For this reason, glycolytic reprogramming should be viewed not simply as one pathway among many, but as an integrative resistance platform. It concentrates multiple processes that are usually treated separately—oncogenic metabolism, immune evasion, stromal adaptation, and treatment failure—into a common biological axis. Recognizing glycolysis as such a convergence point provides a strong conceptual basis for the therapeutic approaches discussed in the next section, particularly those that combine metabolic restriction of tumors with restoration of immune cell fitness. **Figure 4** summarizes how glycolytic reprogramming integrates tumor-intrinsic metabolic adaptation with microenvironmental immune suppression. It also illustrates why glycolysis functions as a shared mechanistic platform underlying immune escape and resistance to radiotherapy, chemotherapy, and immunotherapy.

## 6 Therapeutic opportunities targeting glycolytic immunosuppression

### 6.1 Direct targeting of tumor glycolysis

Given the central role of glycolytic reprogramming in immune escape and therapy resistance, direct targeting of tumor glycolysis has emerged as a rational therapeutic strategy. The goal of this approach is not merely to suppress tumor cell metabolism in a general sense, but more specifically to reduce the metabolic advantages that tumors gain from excessive glucose utilization and to limit the production of immunosuppressive metabolites such as lactate. In principle, interfering with glycolysis may weaken malignant growth while simultaneously helping to re-establish a more immune-permissive microenvironment.

Several nodes within the glycolytic pathway are particularly attractive for intervention. Hexokinase 2 (HK2), lactate dehydrogenase A (LDHA), and pyruvate dehydrogenase kinase 1 (PDHK1) are among the best-characterized regulators that reinforce glycolytic flux and lactate-producing metabolism in tumors (129–131). Inhibition of these molecules has the potential to reduce glucose consumption, restrain pyruvate diversion away from mitochondrial oxidation, and limit extracellular lactate accumulation. Such effects are highly relevant not only for tumor-intrinsic bioenergetics but also for the surrounding immune context, because lowering lactate output and reducing metabolic dominance may alleviate myeloid suppression, improve T-cell fitness, and weaken checkpoint-supportive metabolic states.

Importantly, the rationale for directly targeting glycolysis extends beyond the idea of starving tumor cells. Glycolysis is closely linked to PD-L1 expression, myeloid cell polarization, stromal cooperation, and nutrient competition. Therefore, inhibiting glycolytic pathways may have broader consequences than simple growth suppression. It may partially reverse the ecological conditions that favor immune escape, including lactate-rich immunosuppressive niches, fibroblast-supported exclusionary microenvironments, and glucose-restricted conditions that impair CD8+ T-cell function. This makes direct glycolytic targeting particularly attractive as a component of combination therapy rather than as a purely cytostatic strategy. At the same time, the therapeutic logic of glycolysis inhibition requires precision. The most promising use of this strategy is likely to involve selective disruption of tumor-dominant glycolytic programs while minimizing impairment of beneficial immune cell metabolism. Thus, direct targeting of tumor glycolysis should be viewed as an approach aimed at rebalancing the metabolic ecology of the tumor microenvironment, reducing tumor metabolic privilege while reopening space for effective antitumor immunity.

### 6.2 Nanomedicine and metabolism-directed immunotherapy

Nanomedicine-based strategies have provided an important translational platform for exploiting the link between tumor glycolysis and immune suppression. In this context, the value of nanotechnology lies not simply in drug delivery efficiency, but in its ability to integrate metabolic intervention with immunotherapeutic remodeling. A growing body of work indicates that modulation of glucose metabolism can serve as an immune-sensitizing module, making tumors more responsive to anticancer immunity rather than merely more vulnerable to direct cytotoxic damage.

Studies such as those by Sun and colleagues, Yan and colleagues, and Zhao and colleagues collectively illustrate this principle. Although these platforms differ in design and treatment context, they converge on a shared concept: targeted metabolic modulation can reshape the tumor microenvironment in ways that favor antitumor immunity. In pancreatic cancer, regulation of glucose metabolism through prodrug nanoparticle strategies has been used to enhance photoimmunotherapy, highlighting how metabolic control can improve the immunological consequences of local tumor treatment (132). In other work, metal–phenolic nanomedicines were shown to regulate T-cell antitumor function through a sono-metabolic approach, emphasizing that metabolic intervention can directly support effector immune activity (133). Similarly, glycolysis-targeting nanoplatforms in colorectal cancer have demonstrated that reducing tumor glycolytic activity can help overcome adaptive immune resistance and improve therapeutic responsiveness (134).

What makes these studies especially significant is that they move beyond a simple “drug plus carrier” model. They show that metabolic reprogramming itself can be therapeutically manipulated as a mechanism for improving immune control of tumors. In these settings, nanomedicine is not merely being used to inhibit tumor growth; it is being used to reshape nutrient availability, reduce lactate-associated suppression, attenuate checkpoint-supportive metabolic states, and restore conditions more compatible with T-cell function. This makes metabolism-directed nanotherapy conceptually distinct from conventional chemotherapy delivery. Another strength of nanomedicine is its potential to reduce systemic toxicity and increase local precision, which is particularly important in the context of glycolysis targeting. Because glucose metabolism is also essential for many normal tissues and immune populations, selective delivery becomes crucial. Nanoplatforms may help solve this problem by enriching metabolic modulators in tumors, synchronizing their release with local therapies, or coupling them to immunogenic treatment modalities (135). As a result, nanomedicine offers a practical route to turn metabolic modulation into an immunotherapy-enhancing strategy.

Taken together, these studies support a broader translational message: metabolic control should not be viewed only as a direct anticancer intervention, but also as a way to sensitize tumors to immune attack. Nanomedicine provides one of the most flexible and promising frameworks for implementing this concept *in vivo*.

### 6.3 Nutritional and systemic metabolic intervention

Beyond direct tumor targeting, another emerging strategy involves manipulating host metabolic status or nutrient availability to reshape the tumor microenvironment. This approach is particularly interesting because it expands the scope of cancer immunometabolism beyond tumor cell-autonomous pathways and acknowledges that systemic metabolic conditions can influence local immune responses. Among these strategies, short-term starvation and related metabolic interventions have attracted growing attention for their ability to alter nutrient signaling, stress adaptation, and immune responsiveness in tumors.

Recent work suggests that short-term starvation can enhance anti-PD-L1 therapy by remodeling tumor microenvironment metabolism (136). This observation is important because it indicates that nutritional intervention may not simply slow tumor growth indirectly, but can actively influence the immunometabolic state of the tumor niche. By altering systemic nutrient input and host metabolic signaling, such strategies may reduce tumor metabolic dominance, reshape myeloid and stromal populations, and increase the responsiveness of tumors to checkpoint blockade. In this sense, host metabolic intervention becomes a way of modifying the ecological rules under which tumor and immune cells compete. This line of thinking is especially valuable because it introduces a broader therapeutic perspective. Most reviews of glycolysis and tumor immunity focus primarily on tumor-targeted inhibitors, yet the metabolic environment in which tumors exist is also affected by diet, fasting-associated signaling, endocrine responses, and systemic nutrient allocation (137–140). These variables can influence glycolytic dependency, oxidative compensation, immune cell function, and the composition of the microenvironment. Therefore, nutritional or systemic metabolic modulation may represent an additional layer of therapeutic control that complements direct tumor-targeting approaches. Another important advantage of this strategy is conceptual rather than technical: it highlights that tumor immunosuppression is not solely determined within the cancer cell. Instead, it reflects a dynamic interaction between tumor metabolism, host physiology, and immune adaptability. This creates opportunities to think about treatment more holistically. Rather than viewing immunotherapy resistance only through the lens of tumor mutations or checkpoint signaling, one may also consider whether systemic metabolic states are contributing to an unfavorable local immune niche. Nevertheless, nutritional intervention must be approached with caution. The biological effects of fasting or other systemic interventions may vary according to tumor type, stage, patient nutritional reserve, and treatment context. Even so, this area offers a distinctive and increasingly relevant extension of glycolysis-centered cancer therapy, particularly when the goal is to sensitize tumors to immunotherapy by remodeling the broader metabolic environment in which immune responses occur.

### 6.4 Engineering metabolically competitive immune cells

A complementary therapeutic strategy focuses not on weakening tumor metabolism directly, but on strengthening the metabolic adaptability of immune cells. This approach is especially compelling because it addresses one of the central problems identified throughout this review: in many tumors, immune cells fail not only because they are inhibited, but because they are metabolically outcompeted. Engineering immune cells to better acquire and utilize glucose offers a direct method to improve their survival and function within low-glucose, lactate-rich tumor microenvironments.

Recent studies have provided strong proof of concept for this idea. Overexpression of GLUT1 in CAR-T cells has been shown to enhance glucose uptake, induce favorable metabolic reprogramming, and improve antitumor potency (141). Similarly, GLUT3 overexpression can increase environmental glucose capture and strengthen antitumor efficacy under restrictive conditions (142–144). These findings suggest that improving transporter-mediated glucose access may help therapeutic lymphocytes maintain effector function even when tumor cells dominate nutrient consumption. Rather than accepting the tumor microenvironment as metabolically fixed, these approaches redesign immune cells to function more effectively within it. Engineering glycolytic machinery downstream of glucose uptake offers another promising avenue. HK2-engineered T cells, for example, display increased antitumor activity and can synergize with anti-PD-1 immunotherapy (75). This is particularly important because it links immune cell metabolic reinforcement with checkpoint-based treatment. It implies that restoring or enhancing T-cell bioenergetic competence may allow immune cells to derive greater benefit from the release of inhibitory signaling constraints. In other words, relieving immune inhibition and improving metabolic fitness may be mechanistically complementary rather than redundant. The translational importance of this approach lies in its ability to reverse the logic of nutrient competition. Instead of only attempting to reduce tumor access to glucose, one can also increase the capacity of immune cells to compete successfully for limited nutrients. This is a major conceptual advance in cancer immunometabolism. It suggests that durable antitumor responses may depend not only on suppressing malignant metabolic programs, but also on equipping immune cells with the resilience required to function under metabolic stress. Accordingly, engineering metabolically competitive immune cells may be particularly relevant for solid tumors, where nutrient scarcity and microenvironmental hostility remain major obstacles to adoptive cell therapy. By improving glucose uptake, glycolytic flexibility, and metabolic persistence, such strategies may enhance survival, expansion, and cytotoxicity within the tumor bed. This makes immune-cell metabolic engineering one of the most promising translational extensions of the nutrient competition framework.

### 6.5 Combination strategies and translational challenges

Despite the promise of glycolysis-targeted immunotherapy, several important challenges must be addressed before these strategies can be broadly translated into clinical practice. One major concern is toxicity. Glycolysis is not a tumor-exclusive pathway; it is essential for many normal tissues and is also required by activated immune cells. Systemic suppression of glucose metabolism therefore carries an inherent risk of harming physiological function or unintentionally weakening the very immune responses that therapy is intended to restore (145, 146). This creates a fundamental double-edged effect in which glycolysis is both a driver of tumor progression and a requirement for effective immunity. This issue highlights the need to distinguish between two conceptually different therapeutic strategies. The first aims to suppress tumor glycolysis in order to reduce metabolic dominance, lactate release, and checkpoint-supportive signaling. The second aims to enhance the metabolic adaptability of immune cells so that they can function effectively despite nutrient restriction and metabolic stress. Although these strategies may ultimately be combined, they are not interchangeable. In some contexts, directly inhibiting glycolysis may be beneficial; in others, supporting immune-cell metabolism may be equally or more important. Future therapeutic design will likely need to balance these two directions rather than assuming that all glycolytic activity should be uniformly suppressed.

Another challenge is metabolic heterogeneity across tumor types and even within individual tumors. Not all cancers rely on glycolysis to the same degree, and not all regions of a tumor display identical nutrient gradients or metabolic dependencies. Some tumors may switch between glycolysis and oxidative metabolism, while others maintain mixed metabolic states depending on oxygenation, stromal support, or therapeutic stress. As a result, the efficacy of glycolysis-targeting interventions is unlikely to be universal. Patient selection and metabolic stratification will therefore be essential for identifying those most likely to benefit. This need for precision underscores the importance of biomarkers. Effective translation will require tools that identify glycolysis-high tumors, define lactate-rich or immune-excluded microenvironments, and distinguish tumors in which metabolic suppression is a dominant driver of resistance. Biomarkers may include imaging-based metabolic assessments, expression signatures of glycolytic enzymes and transporters, lactate-related profiles, checkpoint-associated metabolic markers, or indicators of immune cell metabolic fitness (147–149). Without such stratification, glycolysis-targeted interventions risk being applied too broadly or in biologically mismatched settings. Finally, rational combination design will be critical. Because glycolytic immunosuppression operates across tumor cells, immune cells, and stromal compartments, single-agent approaches may have limited durability. The greatest promise likely lies in combination strategies that integrate metabolic restriction of tumors with restoration of immune cell function and checkpoint relief. Such combinations may include glycolysis inhibitors with anti-PD-1/PD-L1 therapy, nanomedicine-guided local metabolic modulation, nutritional interventions that reshape host-tumor metabolic relationships, or adoptive cell therapies engineered for enhanced metabolic resilience (150). The challenge moving forward is not simply to inhibit glycolysis, but to do so in a way that selectively dismantles tumor metabolic privilege while preserving or enhancing antitumor immunity.

In this sense, the future of the field may depend on moving from broad metabolic suppression to ecological precision. The central question is no longer whether glycolysis matters in cancer immunity, but how it can be targeted in a way that accounts for competing cellular demands, tumor heterogeneity, and the dynamic balance between tumor restriction and immune support. Emerging therapeutic strategies targeting glycolytic immunosuppression are summarized in **Table 3**.

**Table 3:** Representative therapeutic strategies targeting glycolytic immunosuppression.

Therapeutic strategy	Study	Cancer type/model	Main therapeutic finding	Translational implication	DOI
Metabolism-directed photoimmunotherapy	Sun F, et al. *Adv Sci (Weinh).* 2021	Pancreatic cancer	Prodrug nanoparticles regulated glucose metabolism and enhanced photoimmunotherapy efficacy	Supports the use of metabolic intervention as an immunotherapy-sensitizing module	10.1002/advs.202002746
Sono-metabolic nanomedicine	Yan J, et al. *ACS Nano.* 2023	Tumor models/T-cell-directed therapy	Metal–phenolic nanomedicines improved T-cell antitumor function through metabolic regulation	Demonstrates that metabolic remodeling can be harnessed to support immune function	10.1021/acsnano.3c02428
Glycolysis-targeted chemo-immunotherapy	Zhao LP, et al., 2024	Colorectal cancer	Inhibition of tumor glycolysis reduced adaptive immune resistance and improved immunotherapeutic efficacy	Supports targeting glycolysis to reverse therapy-associated immune suppression	10.1002/advs.202309204
Nutritional/systemic metabolic intervention	Cheng K, et al., 2025	Hepatocellular carcinoma	Short-term starvation boosted anti-PD-L1 therapy by reshaping tumor microenvironment metabolism	Extends glycolysis-centered therapy from tumor-targeted to host metabolic intervention	10.1097/HEP.0000000000001244
Checkpoint sensitization through glycolysis control	Ding CH, et al. *Cell Death Dis.* 2025	Hepatocellular carcinoma	Targeting the PRMT3/PDHK1/glycolysis axis reduced PD-L1-mediated immune escape	Suggests that glycolysis inhibition may enhance checkpoint-based therapy	10.1038/s41419-025-07482-7
RNA-regulated glycolysis and drug resistance	Ye X, et al. *Front Immunol.* 2024	Hepatocellular carcinoma	IGF2BP1/c-Myc-driven glycolysis promoted immune escape and oxaliplatin resistance	Indicates that glycolysis-associated immune escape is also therapeutically actionable in drug-resistant tumors	10.3389/fimmu.2024.1480834

## 7 Conclusions and future perspectives

### 7.1 Glycolysis should be viewed as an immunological organizer

Taken together, the evidence reviewed here indicates that glycolytic reprogramming should no longer be interpreted solely as a metabolic hallmark of malignant cells. Although its classical significance lies in supporting proliferation, biosynthesis, and stress adaptation, its broader importance is that it actively organizes the immunological architecture of the tumor microenvironment. Through excessive glucose consumption, lactate accumulation, extracellular acidification, and checkpoint-associated signaling, glycolysis shapes how tumor cells, immune cells, and stromal cells interact with one another. In this sense, glycolysis functions not merely as a biochemical pathway, but as a regulatory force that determines whether the tumor ecosystem remains permissive or hostile to antitumor immunity. This perspective has important conceptual consequences. It means that glycolytic metabolism is not simply a parallel feature of tumor progression occurring alongside immune escape. Rather, it is one of the mechanisms through which immune escape is established and maintained. Tumor glycolysis influences myeloid cell polarization, T-cell dysfunction, fibroblast-supported immune exclusion, and the expression of immune checkpoints such as PD-L1. As a result, metabolic rewiring should be considered part of the core machinery of immune suppression rather than an ancillary metabolic adaptation. Recognizing glycolysis as an immunological organizer provides a more integrated framework for understanding how tumor metabolism and tumor immunity co-evolve during progression and treatment.

### 7.2 Nutrient competition is central to ineffective antitumor immunity

A second major conclusion is that nutrient competition must be considered a central determinant of ineffective antitumor immunity. In many tumors, immune failure cannot be fully explained by checkpoint signaling, defective antigen presentation, or altered immune composition alone. Instead, immune cells often operate under persistent conditions of metabolic insufficiency created by highly glycolytic tumor cells. Glucose deprivation, lactate accumulation, and acidification collectively impose a state in which cytotoxic lymphocytes are unable to sustain the metabolic programs required for effective proliferation, cytokine production, and tumor killing. This framework is particularly useful because it helps unify several phenomena that are often discussed separately, including T-cell dysfunction, immune exhaustion, poor response to checkpoint blockade, and suppressive myeloid remodeling. Rather than viewing these as isolated defects, nutrient competition highlights the ecological conditions that connect them. Tumor cells that dominate local nutrient resources gain not only a proliferative advantage but also an immunological one. By restricting substrate availability and generating metabolically hostile conditions, they impose functional constraints on the very immune populations needed for tumor control. This makes nutrient competition not a secondary or supportive concept, but a core explanatory model for why antitumor immunity frequently becomes ineffective in glycolysis-high cancers.

### 7.3 Future therapies should combine tumor metabolic restriction with immune metabolic support

From a translational perspective, the most important implication of this review is that future therapies should move beyond the simple idea of suppressing tumor glycolysis in isolation. Although reducing tumor metabolic dominance remains an attractive goal, durable therapeutic benefit will likely require a dual strategy that combines tumor metabolic restriction with active support of immune cell metabolic fitness. In other words, effective intervention should not only deprive tumors of their glycolytic advantage but also help immune cells function under the resulting metabolic conditions.

Several priorities emerge from this view. First, strategies that restore or preserve T-cell metabolic fitness are likely to be essential, particularly in nutrient-restricted solid tumors. Approaches such as enhancing glucose transport, reinforcing glycolytic resilience, or engineering therapeutic lymphocytes for improved metabolic adaptability may complement checkpoint blockade in a mechanistically meaningful way. Second, future therapies should also aim to remodel suppressive myeloid and stromal compartments, since lactate-driven macrophage polarization and fibroblast-supported immunosuppressive niches are major components of glycolytic immune escape. Third, combination approaches with PD-1/PD-L1 blockade or other immunotherapies are likely to be more effective than standalone metabolic interventions, because checkpoint relief alone may be insufficient if immune cells remain metabolically compromised. At the same time, successful translation will require greater precision in patient selection and biological stratification. Glycolytic dependency varies across tumor types, treatment states, and microenvironmental contexts, and not all patients are equally likely to benefit from glycolysis-centered strategies. Therefore, the development of more refined metabolic subtyping, robust companion biomarkers, and context-specific therapeutic designs will be critical. Future progress in this field will depend on the ability to distinguish tumors driven by glycolytic immune suppression from those in which other metabolic or immunological programs predominate.

In conclusion, glycolytic reprogramming represents a unifying framework that connects tumor metabolism, immune suppression, and therapeutic resistance. Its significance lies not only in fueling tumor growth, but in reorganizing the tumor ecosystem in ways that impair effective immunity. Moving forward, the most promising strategies will likely be those that treat cancer metabolism and cancer immunity as inseparable dimensions of the same biological problem. By combining restriction of tumor metabolic privilege with restoration of immune metabolic competence, next-generation therapies may more effectively convert metabolically hostile tumors into immune-responsive ones.
